# Decreased urinary uromodulin is potentially associated with acute kidney injury: a systematic review and meta-analysis

**DOI:** 10.1186/s40560-021-00584-2

**Published:** 2021-11-15

**Authors:** Ruilian You, Hua Zheng, Lubin Xu, Tiantian Ma, Gang Chen, Peng Xia, Xiaohong Fan, Peili Ji, Li Wang, Limeng Chen

**Affiliations:** 1 Department of Nephrology, Peking Union Medical College Hospital, No 1, Shuaifuyuan, Wangfujing St, Beijing, 100730 China; 2 Department of Epidemiology and Biostatistics, Institute of Basic Medical Sciences, Chinese Academy of Medical Sciences, School of Basic Medicine, Peking Union Medical College, Beijing, 100005 China

**Keywords:** Uromodulin, Acute kidney injury, Biomarkers

## Abstract

**Background:**

Urinary uromodulin (uUMOD) is one of the novel biomarkers for predicting AKI. However, currently available publications showed inconsistent results. We designed this meta-analysis to evaluate the potential association between uUMOD and AKI.

**Methods:**

We searched research articles with no language restriction in Medline, Web of Science, Cochrane Library, Embase, and 3 Chinese datasets from inception to February 2021. We used random-effects models to estimate the standardized mean difference (SMD) between patients with AKI or not, while the leave-one-out method and random-effects meta-regression to evaluate the sensitivity and the impact of potential confounders such as age and surgery.

**Results:**

The meta-analysis comprising 3148 subjects from 11 studies showed that the uUMOD of the AKI group is significantly lower than the non-AKI group (SMD: − 0.71; 95% confidence interval (CI), − 1.00, − 0.42, *P* < 0. 001, *I*^2^ = 78.8%). Subgroup analysis revealed the difference is also significant in a different age, surgery condition, and assay time but not acute rejection (AR) group, especially in children (SMD: − 1.21, 95% CI: − 1.80, − 0.61; *P* < 0.001) and patients undergoing surgery (SMD: − 1.03, 95% CI: − 1.75, − 0.30; *P* < 0.001). Lower uromodulin is associated with higher odds for AKI incidence (odds ratio = 2.47, 95% CI: 1.12, 5.47; *P* < 0.001, *I*^2^ = 89%). Meta-reggression found that age was associated with the SMD of uUMOD. The study outcome was reliably confirmed by the sensitivity analysis.

**Conclusion:**

The present study suggested a negative association between uUMOD and AKI especially in children and surgical patients.

**Supplementary Information:**

The online version contains supplementary material available at 10.1186/s40560-021-00584-2.

## Introduction

Acute kidney injury (AKI) is a collection of syndromes characterized by a sudden decrease in glomerular filtration rate, induced by various causes, like dehydration, sepsis, glomerulonephritis, and acute intoxication. Early recognition can guide clinical management, provide preventive measures, and reduce mortality rates [1]. The most common method of diagnosing AKI is the increase of serum creatinine (Scr) according to various criteria [2]. However, Scr is a late biomarker reflecting the glomerular function. Moreover, Scr provides little reliable information on the prognosis of kidney injury [3]. The lack of sensitive biomarkers is an obstacle to the timely treatment of AKI in the clinical setting. 

Many other biomarkers, such as neutrophil gelatinase-associated lipocalin (NGAL), kidney injury molecule-1 (Kim-1), tissue inhibitor of metalloproteinase-2 (TIMP-2) × insulin-like growth factor-binding protein 7 (IGFBP7), and C–C Motif Chemokine Ligand 14 (CCL14) have been studied for diagnostic value in early AKI detection [4]. However, these biomarkers reflect the cell injury in the setting of AKI, other than the cell function, which can identify a patient’s predisposition and risk of developing AKI. 

Uromodulin is the most abundant protein in human urine, and the daily secretion is about 50–150 mg [5]. It has many biological functions, such as protection against urinary tract infection, regulating water and salt metabolism, immunomodulation, and so on [6]. We could detect urinary uromodulin (uUMOD), circulation uromodulin, and anti-uromodulin antibodies [7]. Recently, uromodulin was reported as a biomarker of the renal tubular reserve function in Fabry nephropathy [8] and could predict tubulointerstitial inflammation in patients with active lupus nephritis [9]. Several studies have also explored the relationship between uromodulin and AKI [10–12]. However, the results among these studies are inconsistent due to their limited sample size. Therefore, we conducted this meta-analysis to evaluate the difference of uromodulin between AKI and non-AKI.

## Methods

### Data sources and searches

We searched the original articles in both the English language database (Pubmed-Medline, Web of Science, Embase and Cochrane library) and Chinese language database (China National Knowledge Infrastructure, Weipu Database, and China Biology Medicine) from inception to February 2021. The searching strategy combined keywords with synonyms in both English and Chinese datasets. The MeSH terms of the Pubmed are "uromodulin" and "acute kidney injury". The free-text terms are "Tamm-Horsfall Protein; Tamm Horsfall Protein; Tamm-Horsfall Glycoprotein; Tamm Horsfall Glycoprotein or Uromucoid" and the freedom combination of "acute, kidney, renal, injury, insufficiency, and failure". The syntax used for the English database is shown in Additional file 1: Tables S1–S4. There was no restriction on publication forms or language. Besides, we manually searched the reference lists to find relevant studies.

### Study selection

We screened all original articles with the inclusion criteria: (1) studies included AKI patients and non-AKI group; (2) uUMOD was detected and its relationship with AKI was discussed. Exclusion criteria were as follows: (1) manuscripts in the format of notes, letters, editorials, conference posters, or reviews; (2) studies without specific data of the uUMOD. 

### Data extraction and quality assessment

Two independent investigators screened all retrieved records according to the titles and abstracts and resolved the discrepancies by discussion. For the manuscripts without specific data, we sent e-mails to authors requesting original data. The extracted study characteristics were countries of origin, publication years, study types, ages, inclusion objects, conditions in renal disease, the AKI definition, the measurement-related data (assay method, time, and measurement object). We collected the outcomes information, including AKI occurrence, AKI recovery, renal replacement therapy, mortality, the in-hospital time, and cost. 

We evaluated the quality of the studies by the non-randomized studies of interventions (ROBINS-I) tool [13]. This tool includes seven confounding biases, namely bias due to confounding; bias in the selection of study participants; bias in exposure measurement; bias due to misclassification of exposure during follow-up; bias due to missing data; bias in the measurement of outcomes; and bias in the selection of reported results. The levels of all bias were stratified as "low", "moderate", "serious", and "critical". The results were visualized by the web-based application robvis (https://mcguinlu.shinyapps.io/robvis/) [14].

### Data synthesis and analysis

The statistical analyses were performed with STATA (Version 12; StataCorp, College Station, Texas) and R 4.0.2 software. Data presented as the median and interquartile range (IQR) or range, we converted data to mean and standard deviation (SD) according to the Wan formula [15]. Where data were presented in geometric mean and 95% confidence interval (CI), we transformed it to the arithmetic mean and SD according to the Higgins formula [16]. The differences in pooled standardized mean difference (SMD) of uUMOD between AKI and non-AKI in subgroups (divided by age and surgery condition) were analyzed. Followed the "patients (patients who are at the risk of developing AKI), intervention (concentration levels of uUMOD), comparison (non-AKI patients), and outcomes (the incidence of AKI) (PICO)" principles, we extracted the hazard ratio (HR) and odds ratio (OR) of included studies. 

We synthesized the studies by fixed-effects models with low heterogeneity (*I*^2^ < 50%), otherwise a random-effects model will be performed. Subgroup difference analysis was conducted by Revman 5.3 version (The Cochrane Collaboration, Oxford, UK). The heterogeneity analysis was performed by Cochrane's *Q* test (significance level of *P* < 0.10) and *I*^2^ statistics (ranges from 0 to 100% with lower values representing less heterogeneity), while further analysis of the heterogeneous source by Galbraith radial plot, and the leave-one-out method for sensitivity analysis. Meta-regression was made in the restricted maximum likelihood (ReML) model to check if the age, surgery conditions, and assay time are the sources of the heterogeneity (*P* ≤ 0.05 was considered significant). 

## Results

### Study selection and characteristics

**Fig. 1:**
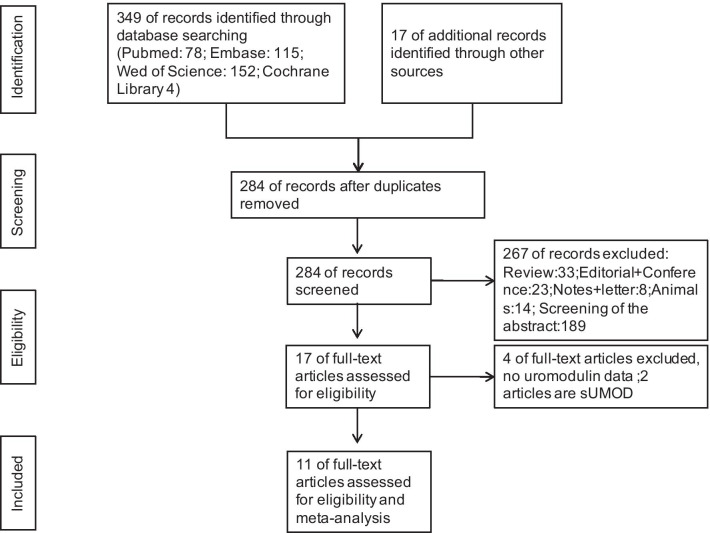
Search plot diagram. Additional records include articles searching from the Chinese language database. *sUMOD* serum uromodulin

As shown in Fig. 1, there are 15 studies focused on the relationship between uUMOD and AKI, but 4 studies without specific uUMOD data [10, 17–19]. A total of 11 studies were included in the meta-analysis [20–30]. Except for one paper written in German, others were written in English. Three studies investigated the relationship between uUMOD and surgery-associated AKI, while two studies recruited participants undergoing renal transplant surgery. The patients in five studies are below 18 years old, with infants and newborns in three studies. Five studies were case–control studies, while six studies were cohort studies. The study "Ashwani (2017)" listed the causes of AKI as pre-renal AKI (*n* = 22), acute tubular necrosis (ATN) (*n* = 3), and hepatorenal syndrome (HRS) (*n* = 12). And it reported there was no difference in the uUMOD/creatinine concentration among the different causes. The causes for AKI in study "P. Jeremy (1993)" were acute rejection (AR) (*n* = 37) and ATN (*n* = 7). As for the outcome information, only *"Ashwani* *(2017)"* offered the concentration of uUMOD and renal function recovery after the liver transplant surgery and suggested there was no significant correlation. The main characteristics of the studies are shown in Table 1.

**Table 1:** Characteristics of the studies included in the meta-analysis

Reference (Year)	Country	Study type	Age	AKI definition Increase in	Measurement	Methods	Assay time	Inclusion patients	Renal disease of Non-AKI group
Ashwani [24] (2017)	USA	Prospective retrospective cohort study	56 (50–62)	Scr > 50% and/or > 0.3 mg/dL from baseline within 3 m	uUMOD/Cr	MSD	During the AKI	Adult patients with liver cirrhosis listed for LT	Partly
Askenazi [12] (2012)	USA	Nested case–control study	Newborns	Scr > 0.3 mg/dL within 48 h or a persistent rise in Scr to ≥ 1.7 mg/dL within 3 days after birth	uUMOD	MSD	The first 4 days	Neonatal intensive care unit	ND
Askenazi [21] (2016)	USA	Cohort study	Infants	KIDGO	uUMOD	MSD	first 4 days	VLBW infants	ND
Michael [19] (2018)	USA	prospective study	Non-AKI 3.9 (0.7–6.6); AKI 1.8(0.5–5.3)	Scr > 50%pre-procedural baseline within no 48 h, post-surgery + modified KDIGO	uUMOD	ELISA	Before LT surgery	Patients < 18 years of age undergoing cardiac surgery with CPB	ND
Sweetman [20] (2016)	Ireland	Cohort study	Infants	Scr-based neonatal modification of 2012 KDIGO	uUMOD	MSD	Exposed to PA on days 1, 2, 3 and 7 of life	Infants exposed to perinatal asphyxia	ND
Bullen [23] (2019)	USA	Cohort study	Non-AKI 73(9), AKI 74(10)	ND	uUMOD	MSD	Baseline	Systolic BP Intervention Trial subjects	CKD
Romero [27] (2002)	Argentina	Case–control trial	Non-AKI 31(16–60); AKI 40(19–67)	Scr to values > 130umol/L	24 h-uUMOD	ELISA	After surgery	Liver transplant patients	ND
Dehne [29] (1998)	Germany	Case–control trial	AKI (36.3 ± 9.8); Non-AKI (37.7 ± 16.9)	ND	24 h-uUMOD	ELISA	The begining five days of AKI	Intensive care unit patients	ND
Jeremy [28] (1993)	UK	Case–control trial	Adults	ATN was defined by a rise in serum creatinine level in the absence of histological evidence of rejection in an allograft biopsy. AR: diagnosed on the basis of physical findings, renal functional parameters and allograft biopsy	uUMOD	ELISA	After surgery	Patients in end-stage renal failure who received kidney transplants	ESRD
Pranav S [25] (2017)	USA	Cohort study	68.1 ± 11.3	Scr ≥ 0.3 mg/dl or .1.5 times the preoperative value during the first 72 h after surgery	uUMOD/Cr	ELISA	Before surgery	Adults scheduled to undergo on–pump cardiac surgery	ND
Tara K [26] (2010)	USA	Case–control trial	AR 12 ± 5; Non-AKI 14 ± 5	AR: diagnosed by the Banff criteria	uUMOD	ELISA	After surgery	Patients who received kidney transplants	ND

*AKI* acute kidney injury, *AR* acute rejection, *ATN* acute tubular necrosis, *CKD* chronic kidney disease, *ELISA* enzyme linked immunosorbent assay, *IQR* interquartile range, *KIDIGO* kidney disease improving global outcomes, *MSD* meso scale discovery, *ND* no data, *Scr* serum creatinine, *uUMOD* urinary uromodulin, *VLBW* very low birth weight

### Quality assessment and publication bias

**Fig. 2:**
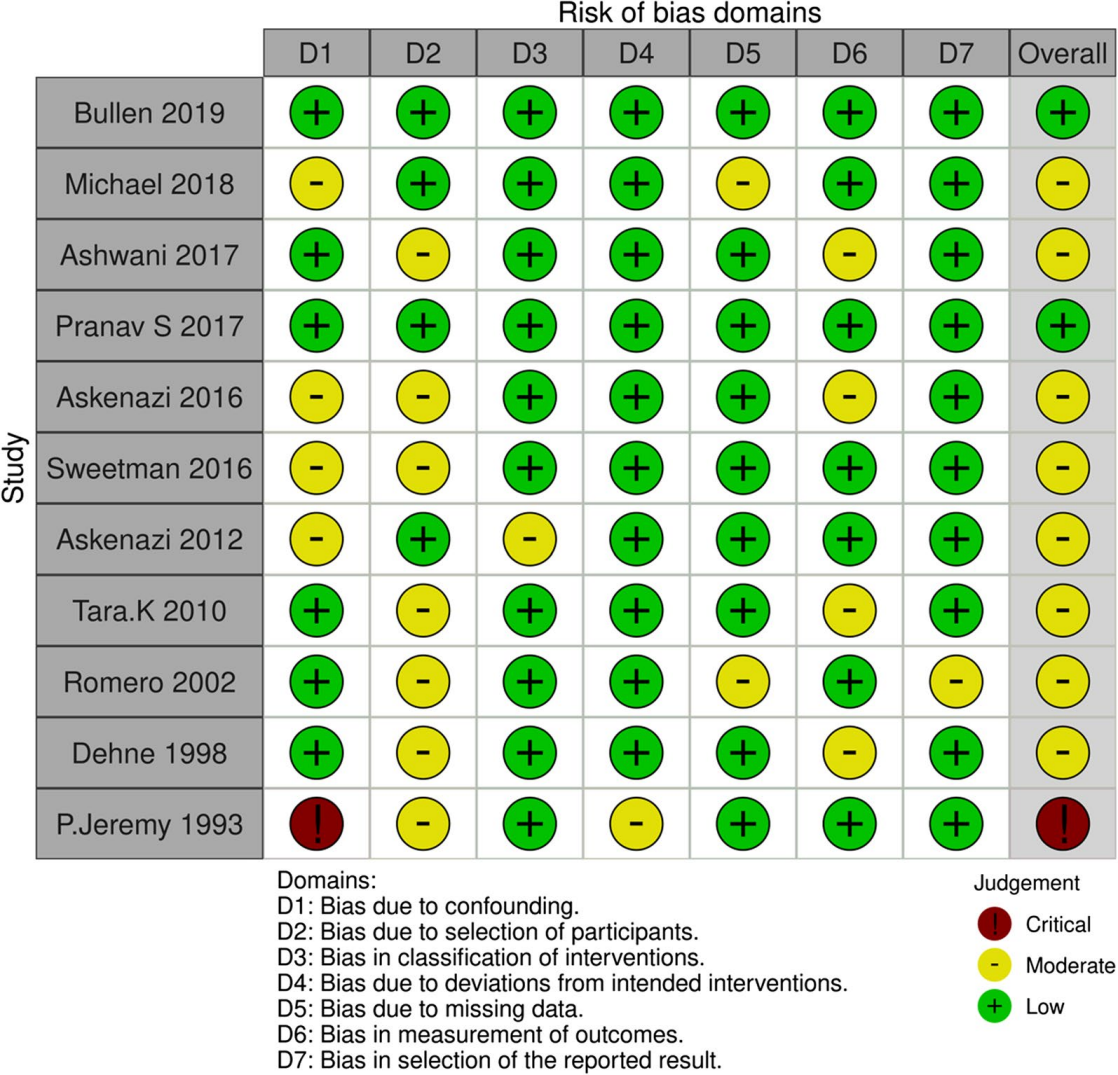
Risk of bias for included studies

Based on the ROBINS-I tool, 2 studies were identified as "low risk", while 8 articles were assessed as "moderate risk" studies. One study was considered as "critical risk" The details are shown in Fig. 2. The Egger's test indicated no publication bias in this meta-analysis (*P* = 0.213) (Additional file 1: Fig. S1).

### Quantitative data synthesis

In total, the sample size of this meta-analysis was 3148 subjects, with 468 AKI patients, and 2680 non-AKI patients. The detailed mean and SD of the uUMOD are in Additional file 1: Table S5. Because of the high heterogeneity of the included studies, the meta-analysis was carried out by the random-effects model. The SMD of uUMOD was significantly lower in the AKI group (− 0.71; 95% confidence interval (CI): − 1.00, − 0.42; *P* < 0.001) than the non-AKI counterparts.

**Fig. 3:**
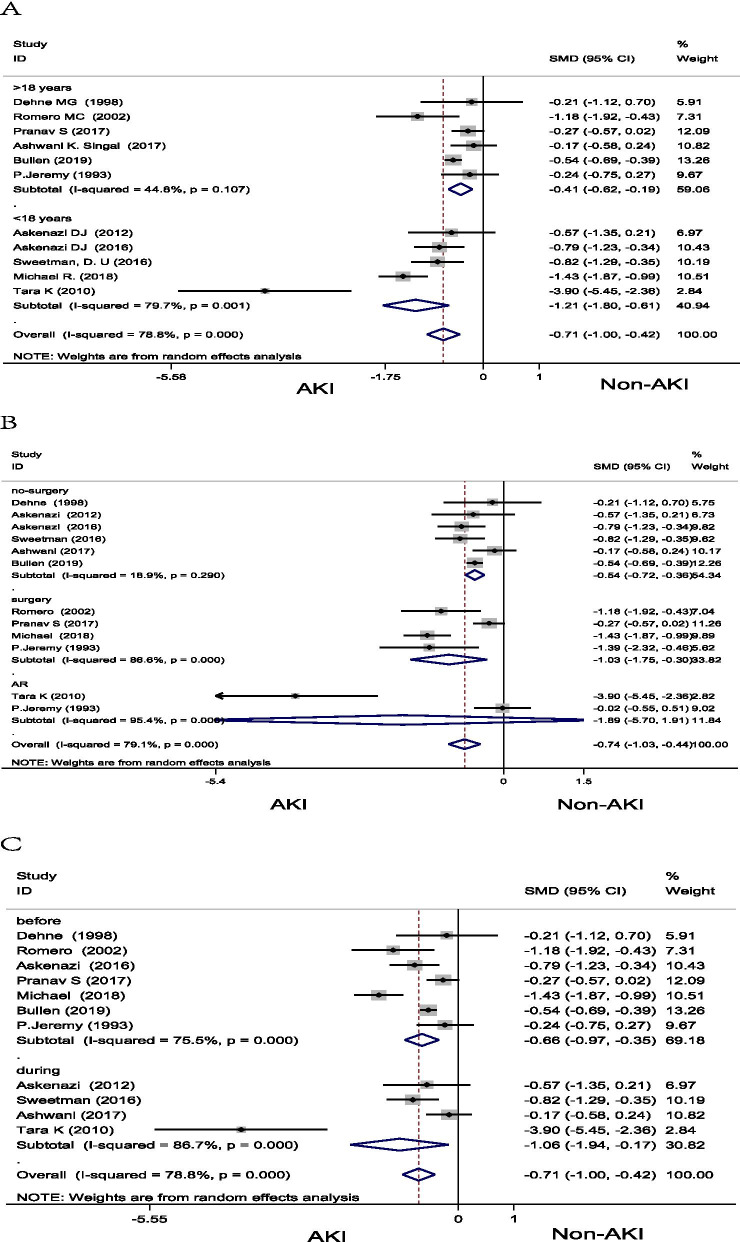
Subgroup analysis of the difference between urinary uromodulin in the patients of AKI and non-AKI according to age (**A**), surgery condition (**B**) and assay time (**C**). *AKI* acute kidney injury, *AR* acute rejection, *CI* confident interval, *SMD* standardized mean difference

We conducted subgroup analysis according to age, surgery conditions, and assay time. Subgroup analysis by age showed that the uUMOD was significantly lower in the AKI group in both age > 18 years old (SMD = − 0.41, 95% CI: − 0.62, − 0.19; *P* < 0.001) and age < 18 years old (SMD = − 1.21, 95% CI: − 1.80, − 0.61; *P* < 0.001) (Fig. 3A). When stratified by surgery conditions, the uUMOD was significantly lower in the AKI group in "surgery" (SMD = − 1.03, 95% CI: − 1.75, − 0.30; *P* = 0.005) and "no-surgery" groups (SMD = -0.54, 95% CI: − 0.72, − 0.36; *P* < 0.001), while no significant difference in the AR group (SMD = − 1.89, 95% CI: − 5.70, − 1.91; *P* = 0.329) (Fig. 3B). In the subgroup analysis of assay time, the concentration of uUMOD is significantly different between AKI and non-AKI in “before” (SMD = − 0.66, 95%CI: − 0.97, − 0.35; *P* < 0.001) and “during” subgroups (SMD = − 1.06, 95%CI: − 1.94, − 0.17; *P* = 0.019) (Fig. 3C). The difference of the uUMOD between AKI and non-AKI is more significant in younger age, as the subgroup difference analysis in age showed the *P* is 0.004, while surgery conditions is 0.36, and assay time is 0.44.

**Fig. 4:**
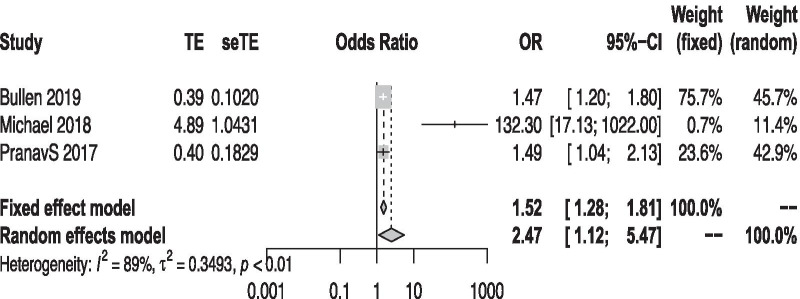
A forest plot of incidence of acute kidney injury. *CI* confident interval, *OR* odds ratio

We pooled the hazard ratio (HR) and odds ratio (OR) of three included studies [20, 24, 26]. We converted the reciprocal HR to take the high-level uromodulin as control [31]. Randomized meta-analysis showed the lower uromodulin is associated with the increased risk of developing AKI (pooled OR: 2.47, 95% CI: 1.12, 5.47; *P* < 0.001; *I*^2^ = 89.2%) (Fig. 4).

### Heterogeneity analysis and sensitivity analysis

We detected moderate heterogeneity for all included studies (*I*^2^ = 79.5%, *P* < 0.001). For further analysis on finding the heterogeneous source, the Galbraith radial plot showed studies "Michael (2018)" and "Tara K (2010)" were fallen outside the 95% CI line, while studies "Pranav S (2017)" and "Ashwani (2017)" are on the 95% CI line (Additional file 1: Fig. S2). The *I*^2^ reduced to 38.3% and *P* = 0.113 when removed "Michael (2018)" and "TaraK (2010)" studies. Subgroup analysis of age showed that the *I*^2^ are 44.8% (> 18 years) and 79.7% ("< 18 years" group), while *P* = 0.107 (" > 18 years" group) and 0.001 (" < 18 years" group). Subgroup analysis of surgery showed the *I*^2^ = 18.9% ("no surgery" group), 90.2% ("surgery" group) and 94.5% ("AR*"* group), while *P* = 0.290 ("no surgery" group) and *P* < 0.001 in "surgery" and "AR" subgroups. The *I*^2^ are 75.5% and 86.7% in the "before" and "during" subgroups, respectively, and the *P* values are less than 0.001 in the two groups. 

The leave-one-out method showed the result of the pooled SMD and OR is robust (Additional file 1: Figs. S3, S4). Meta-regression analysis showed that age was one source of the heterogeneity (*P* = 0.079) (Additional file 1: Fig. S5). Surgery conditions and assay time were not the sources of the heterogeneity (*P* > 0.1).

## Discussion

In this study, we first validated that uromodulin could diagnose and predict AKI by meta-analysis. We observed that uUMOD in the AKI group was significantly lower than the non-AKI group. Subgroup analysis showed that the difference of uromodulin between AKI and non-AKI was more significant in children than in adults, and could predict AKI before the operation. 

AKI occurs in approximately 10–15% of in-hospital patients, while more than 50% in intensive care units [32]. It elevates mortality rates, extends hospitalization, and increases expenses [33]. In the past 50 years, the diagnosis of AKI was mainly based on Scr [34], which was influenced by muscle mass, diet, age, medication, hydration status, and sex. Besides, for most forms of AKI, the renal tubular epithelium is the primary locus of injury, not glomeruli reflected by Scr. Decreased eGFR based on the Scr is a late and insensitive indicator that cannot timely reveal the kidney injury within 25–50% of renal function lost, thus far from sufficient in clinical practice [35]. In the end, there is no optimal biomarker in the prediction or diagnosis of AKI, with a promising direction for machine learning and artificial intelligence in the construction of a clinical prediction model utilizing a set of potential biomarkers. Thus, the discovery of more biomarkers is meaningful.

Uromodulin is exclusively produced by the cells in the thick ascending limb (TAL) and the distal convoluted tubule (DCT) [36]. Though extensive biochemical studies have been conducted, the physiological roles of uromodulin remained elusive for decades. Genome-wide association studies (GWAS) identified that common UMOD variants were strongly associated with renal function and the chronic kidney disease (CKD) progress risk in the general population [37, 38]. Uromodulin has regained the researcher's attention. Studies showed that uromodulin was correlated with various diseases, including hypertension, CKD, diabetic nephropathy [39], and diabetic-related aortic stiffness [40].

Several studies have elucidated that uromodulin can protect against AKI in various ways. LaFavers KA reported that uromodulin could protect against AKI by suppressing the activity of transient receptor potential cation channel, subfamily M, member 2 (TRPM2), a multifunctional nonselective calcium ion channel, attenuating systemic oxidative stress via interfering with the RAC1/JNK/c-JUN pathway [41]. THP-deficient (THP^−/−^) mice have more tubular casts, increased inflammatory reaction and necrosis, and worse renal function. THP may exert protective function by raising the expression of TLR4 and its targeting to the apical membrane of proximal tubular S3 segments, where the interaction of TLR4 with pro-inflammatory interstitial ligands is released after ischemia [42]. Complement activation was a reported cause of renal ischemia–reperfusion injury [43]. Recently collectin-11 (CL-11) was considered to be involved in AKI, and uromodulin could bind to the CL-11 mediated by the glycan, dampening the lectin pathway [44]. After ischemic AKI, UMOD was redistributed in the TAL with the presence of interstitial increasing, which would decrease the transition of macrophages from a pro-inflammatory (M1) to a pro-healing (M2) phenotype [45]. This process could positively regulate mononuclear phagocyte numbers, plasticity, and phagocytic activity. Thus inhibit the persistent inflammatory response and enhancing kidney recovery after ischemia–reperfusion injury [46]. The detailed underlying mechanism of the relationship between AKI and UMOD needs further investigation. 

Our study further validated that uromodulin could be a potential biomarker to diagnose and predict AKI. As a candidate for building a diagnostic model and early risk stratification model of AKI, it could improve the efficacy. As for diagnosing AKI, the area under the curve (AUC) of the receiver operating characteristic curve (ROC) of the study "Michael 2018" is 0.9 [20], while in the study "Askenazi 2012" is 0.77 [23]*.* For predicting AKI, studies revealed that the lowest quartile of uUMOD is associated with an increased risk of developing AKI compared with the highest quartile. In 2019, Bullen demonstrated that lower uromodulin independently predicted subsequent AKI, adjusted by eGFR and albuminuria [24]. 

Subgroup analysis of different ages showed the uUMOD was significantly lower in the AKI group. Compared with the adults, Scr was not considered good enough to predict AKI in children at the growing stage, whose muscle volume kept increasing. The baseline Scr concentration in younger children is 0.2–0.3 mg/dl, which is close to the laboratory variability of 0.1–0.2 mg/dl. Therefore, an absolute increase in Scr might vary substantially in children and was not suitable for diagnosing AKI in younger ages [47]. The serum UMOD levels also increased significantly during growth [48], but with the elucidated subgroup difference in different age groups. Therefore, uUMOD is more valuable and helpful to predict AKI of children, as well as a more easy to accessible and non-invasive process than serum UMOD for children. Intensively, since children have fewer complications than adult patients, there are fewer confounding variables in the outcome, enhancing the potential diagnostic efficacy.

Another subgroup analysis revealed the consistent result of the uUMOD in AKI prediction of surgery groups. AKI is a common postoperative complication, with the incidence is about 8% in general surgery and about 30% in cardiac surgery [49]. Approximately 80% of AKI cases in surgery were due to ischemia that occurred during an operation or postoperative blooding [50], which caused fluid depletion and renal hypofusion, activating adaptive mechanisms to maintain normal function. GFR is initially decreased without renal injury and continue to cause organ damage when the hypoperfusion sustained, or the adaptive response was not adequate. It always happened because of the autoregulation mechanism disorder, the sympathetic nervous system and renin–angiotensin–aldosterone system (RAAS) therapy. If the kidney could not restore adequate perfusion, continuous organ damage and nutrient deficiency and ATP-depletion would lead to the necrosis or apoptosis of the epithelial cell, activation of the inflammatory response and parenchymal damage [51]. Early recognition of AKI caused by hypovolemia is quite important since it is relatively easier to handle compared with other AKI causes. However, currently, few biomarkers can specifically predict surgery-associated AKI. Pranav S reported that preoperative uUMOD could predict AKI developing after cardiac surgery, as well as liver transplantation in Ashwani's study [25, 26]. Our study provided further evidence for the promising application of uUMOD in predicting operation-associated AKI, which was mostly caused by circulatory volume reduction and could be preventable. 

The assay time subgroup analysis shows that the difference of uUMOD between AKI and non-AKI is significant in both "before" and "during" subgroups, indicating that uUMOD can predict and detect AKI. The pooled OR suggested the lowest quantile uromodulin at baseline is 2.47 times of developing AKI than the highest quantiles. With the limited data of the included studies, we considered it is better to send UMOD when people are at the risk of developing AKI, such as septic or perioperative patients. What's more, sequential measurements of uUMOD are needed to help define the optimal timing of sampling and assessing its utility as a marker of detective, predictive, and recovery from AKI.

Uromodulin has some advantages in predicting and diagnosing AKI. The pathological analysis of AKI showed tubular injuries were in the outer renal medulla, the late proximal tubule (S3), and the TAL, the primary secretion site of uromodulin. UMOD could reflect the reserve of the kidney function. The other well-known AKI markers, such as NGAL, TIMP-2/IGFBP7, were widely used to predict the acute injury and recovery of the renal tubular for years. But NGAL is expressed mainly in the distal tubular and induced in injured epithelia [52], the TIMP-2 secreted in the distal tubule, while IGFBP7 is from the proximal tubule [53]. None of them was good enough to predict all AKI patients in critical patients or with low risk. Secondly, once injury has occurred, those well-known markers to forecast AKI are ineffective [32]. The uromodulin did quite well in predicting early AKI in children, in patients with septic or perioperative surgery. However, the development of a biomarker includes five phases, namely discovery, quantification/verification, validation, and implementation [54]. uUMOD is currently at the discovery phase, and future large-scale multicenter prospective studies are needed to precisely evaluate the diagnostic efficacy and cut-offs of uUMOD and to determine the range of its concentration in people with and without AKI. The comparison of sUMOD and uUMOD could find out which would be a better indicator [55].

Our study has some strengths. To our knowledge, itis the first study that pooled the existed studies focusing on the UMOD and AKI. We conducted the subgroup analysis on age, surgery condition, and assay time. Meta-regression analysis focused on the age, surgery condition, and assay time screened that age might be a confounder. We found that the uUMOD played an important role in predicting and diagnosing the AKI, especially in children and patients before surgery. We combined the OR and find that the lower baseline uromodulin is inversely associated with the AKI occurrence. The outcome of the meta-analysis is robust: The heterogeneity of the studies was mainly sourced from the studies "Michael (2018)" and "Tara K (2010)", with the *I*^2^ reduced to 46% (*P* = 0.07). It was partially attributed to the confounding factors and has been balanced by subgroup analysis. 

There are several limitations to the present study. First, the included studies did not serve the sensitivity or specificity of uUMOD, we could not do the diagnostic meta-analysis and get the synthetic cut-off value, sensitivity, or specificity of uUMOD for predicting AKI. Second, the vast majority of studies originated from Western countries; thus, extrapolation of these results to Eastern populations is questionable. Third, we completed the analysis before pre-registration without PROSPERO registration number, which should be avoided in our future meta-analysis. Fourth, several studies did not offer the causes of AKI, so we could not make the subgroup analysis of different causes of the AKI. Fifth, only one animal study detected the concentration of the uUMOD without specific data [56]. Two studies offered the fold-change of the UMOD mRNA levels in mice kidney tissue [57, 58]. So we did not combine the SMD of the uUMOD. Last, we pooled the HR and OR together which may cause some bias.

In summary, this study is the first systematic review and meta-analysis combining current manuscripts in the field on the utility of uromodulin in the prediction of AKI. We observed that decreased uUMOD might be a potential novel biomarker for AKI prediction, especially in children and patients before surgery. 

## Supplementary Information

**Additional file 1: Table S1.** Search strategy for PubMed. **Table S2.** Search strategy for Embase. **Table S3.** Search strategy for Cochrane Library. **Table S4.** Search strategy for Web of Science. **Table S5.** Mean and standard deviations of the uUMOD included in the meta-analysis. **Fig. S1.** Egger plot shows detailing publication bias in the included studies. **Fig. S2.** Galbraith radial plot of Heterogeneity analysis. **Fig. S3.** Sensitivity analysis to test the robustness of the pooled Standardized Mean Difference by leave-one-out method. **Fig. S4.** Sensitivity analysis to test the robustness of the pooled Odds Ratio by leave-one-out method. **Fig. S5**. Meta-regression plot of the association between SMD of the uUMOD with age. MOOSE checklist.

## Data Availability

All data generated or analyzed during this study are included in this published article. Corresponding author can be contacted for more information.

## Abbreviations

AKI: Acute kidney injury
AR: Acute rejection
ATN: Acute tubular necrosis
AUC: Area under the curve
CCL14: C–C motif chemokine ligand 14
CI: Confidence interval
CKD: Chronic kidney disease
CL-11: Collectin-11
DCT: Distal convoluted tubule
GFR: Glomerular filtration rate
GWAS: Genome-wide association study
IGFBP7: Insulin-like growth factor-binding protein 7
IQR: Interquartile range
Kim-1: Kidney injury molecule-1
NGAL: Neutrophil gelatinase-associated lipocalin
OR: Odds ratio
RAAS: Renin–Angiotensin–Aldosterone-System
ReML: Restricted maximum likelihood
ROC: Receiver operating characteristic curve
SD: Standard deviation
SMD: Standardized mean difference
TAL: Thick ascending limb
THP: Tamm-Horsfall protein
TIMP-2: Tissue inhibitor of metalloproteinase-2
TRPM2: Transient receptor potential cation channel, subfamily M, member 2
UMOD: Uromodulin
WT: Wild type
