# Supplementary material for: Decreased urinary uromodulin is potentially associated with acute kidney injury: a systematic review and meta-analysis
Source: J Intensive Care. 2021 Nov 15;9:70. doi: 10.1186/s40560-021-00584-2 (PMC8591828; doi:10.1186/s40560-021-00584-2)
Supplement: Supplementary file 1 — Additional file 1: Table S1. Search strategy for PubMed. Table S2. Search strategy for Embase. Table S3. Search strategy for Cochrane Library. Table S4. Search strategy for Web of Science. Table S5. Mean and standard deviations of the uUMOD included in the meta-analysis. Fig. S1. Egger plot shows detailing publication bias in the included studies. Fig. S2. Galbraith radial plot of Heterogeneity analysis. Fig. S3. Sensitivity analysis to test the robustness of the pooled Standardized Mean Difference by leave-one-out method. Fig. S4. Sensitivity analysis to test the robustness of the pooled Odds Ratio by leave-one-out method. Fig. S5. Meta-regression plot of the association between SMD of the uUMOD with age. MOOSE checklist. [file 40560_2021_584_MOESM1_ESM.docx]

**Additional Materials:**

1. **Table S1 Search strategy for PubMed**
2. **Table S2 Search strategy for Embase**
3. **Table S3 Search strategy for Cochrane Library**
4. **Table S4 Search strategy for Web of Science**
5. **Table S5. Mean and standard deviations of the uUMOD included in the meta-analysis.**
6. **Figure S1. Egger plot shows detailing publication bias in the included studies.**
7. **Figure S2. Galbraith radial plot of Heterogeneity analysis.**
8. **Figure S3. Sensitivity analysis to test the robustness of the pooled Standardized Mean Difference by leave-one-out method.**
9. **Figure S4. Sensitivity analysis to test the robustness of the**

**pooled Odds Ratio by leave-one-out method.**

1. **Figure S5. Meta-regression plot of the association between SMD of the uUMOD with age.**
2. **MOOSE checklist**

**Table S1. Search strategy for PubMed.**

**Table S2. Search strategy for Embase**

**Table S3. Search strategy for Cochrane Library**

**Table S4. Search strategy for Web of Science**

**Table S5. Mean and standard deviations of the uUMOD included in the meta-analysis.**

| **Reference（Year)** | **Unit of uromodulin** | **AKI**  **number** | | **AKI**  **mean** | | **AKI**  **SD** | | **Non-AKI**  **number** | | **Non-AKI**  **mean** | | **Non-AKI**  **SD** |  |
| --- | --- | --- | --- | --- | --- | --- | --- | --- | --- | --- | --- | --- | --- |
| Ashwani（2017） | pg/mg creatinine | 32 | 2.40x10^6^ | | 2.43x10^6^ | | 79 | | 2.92x10^6^ | | 3.25x10^6^ | | |
| Askenazi（2012） | pg/ml | 9 | 15.96 | | 16.7 | | 24 | | 42.1 | | 52.6 | | |
| Askenazi（2016） | pg/ml | 27 | 5.11x10^5^ | | 3.46x10^5^ | | 84 | | 8.58x10^5^ | | 4.66x10^5^ | | |
| Michael（2018） | μg/ml | 47 | 4.56 | | 3.08 | | 54 | | 14.57 | | 9.12 | | |
| Sweetman（2016） | pg/ml | 30 | 1.22x10^6^ | | 6.57x10^6^ | | 52 | | 2.52x10^6^ | | 1.93x10^6^ | | |
| Bullen（2019） | pg/ml | 184 | 5330 | | 3736 | | 2167 | | 7330 | | 3709 | | |
| Romero （2002） | mg/24h | 14 | 36.57 | | 29.82 | | 20 | | 97.33 | | 62.39 | | |
| Dehne （1998） | mg/24h | 7 | 13.4 | | 5.2 | | 14 | | 15.5 | | 11.6 | | |
| Pranav S  (2017) | ug/g | 64 | 9.97 | | 11.07 | | 154 | | 13.83 | | 15.12 | | |
| P.Jeremy  (1993) | μg/ml | 44 | 8.4 | | 6.09 | | 22 | | 9.88 | | 6.25 | | |
| Tara K（2010） | μg/ml | 10.00 | 5.50 | | 0.85 | | 10.00 | | 13.95 | | 2.94 | | |

Abbreviations: *SD* Standard Deviation, *AKI* Acute Kidney Injury, *uUMOD* urinary uromodulin


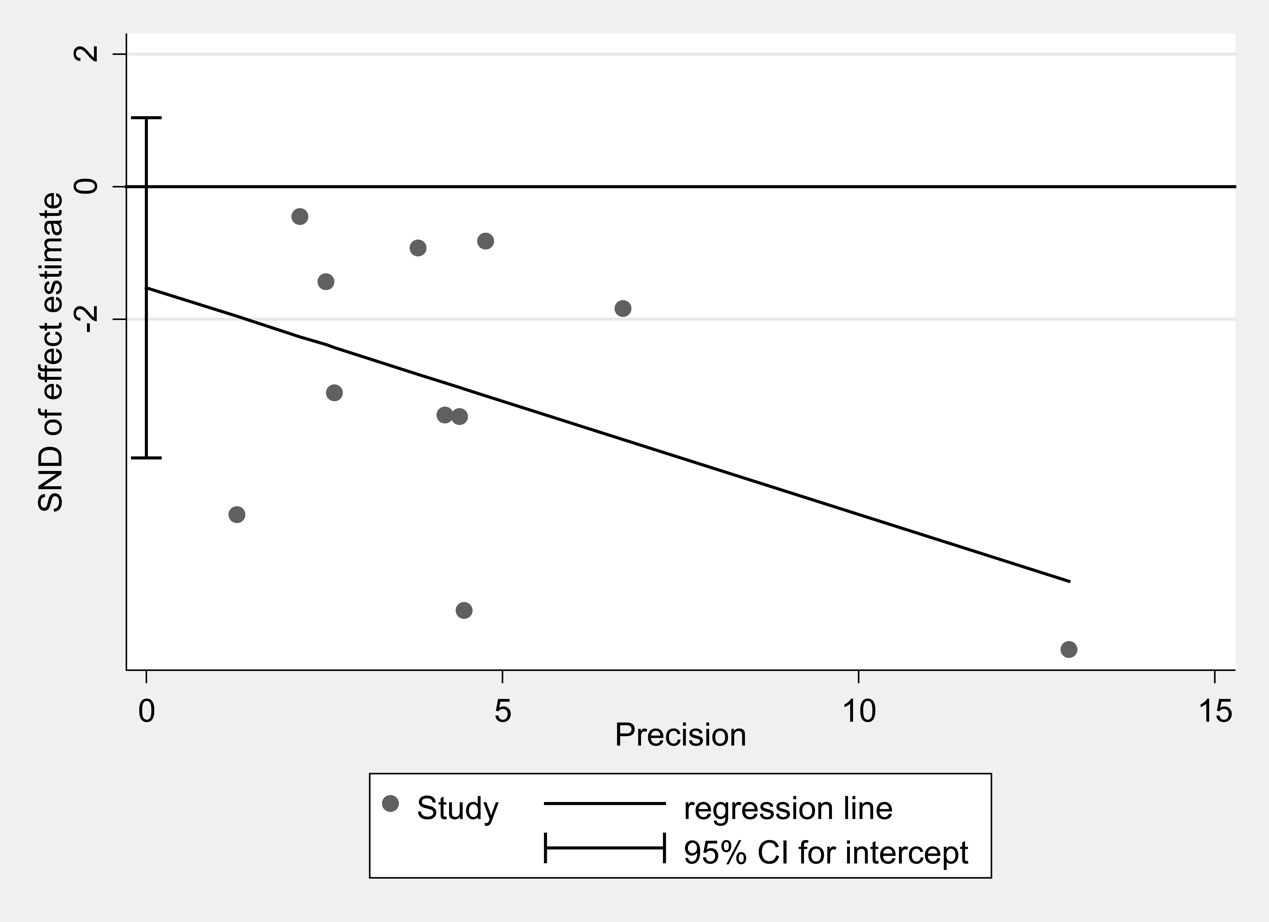


**Figure S1. Egger plot shows detailed publication bias in the included studies.** No small-study effect was found (*P* = 0.213).

**Figure S2. Galbraith radial plot of Heterogeneity analysis.**The graph shows the Study "*Michael (2018)*" and "*Tara K (2010)*" are out of the 95% confidence interval (CI) line, "*Ashwani (2017)*" and "*Pranav S (2017)*"are on the line.

 **Figure S3. Sensitivity analysis to test the robustness of the pooled Standardized Mean Difference by leave-one-out method.** The result wasn’t affected significantly after deletion of any study.

**Figure S4. Sensitivity analysis to test the robustness of the pooled Odds Ratio by leave-one-out method.** The result of meta-analysis of the odds ratio was robust.


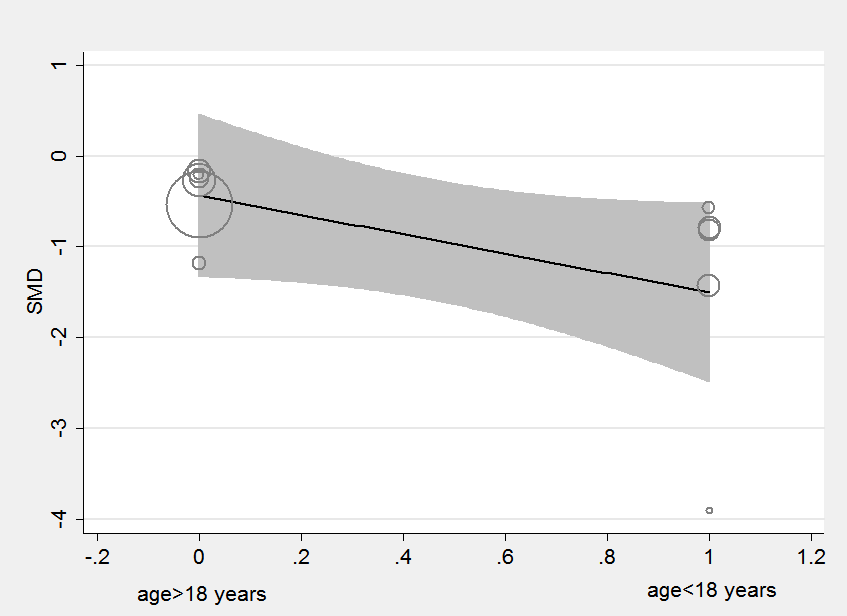


**Figure S5. Meta-regression plot of the association between SMD of the uUMOD with age.**A significant relationship was revealed as *P* = 0.079 (*P* < 0.1 considered as significant).

Abbreviations: *SMD* Standardized Mean Difference, *uUMOD* urinary uromodulin.

**MOOSE checklist**

| **Reporting of background should include** | |
| --- | --- |
| Problem definition | Background |
| Hypothesis statement | Background |
| Description of study outcome(s) | Overall survival, metastasis value |
| Type of exposure or intervention used | Acute kidney injury |
| Type of study designs used | Meta-analysis |
| Study population | Global |
| **Reporting of search strategy should include** | |
| Qualifications of searchers (eg, librarians and investigators) | Investigator |
| Search strategy, including time period included in the synthesis and keywords | Data Sources and Searches |
| Effort to include all available studies, including contact with authors | We contact authors and searched reference lists and citations |
| Databases and registries searched | Methods |
| Search software used, name and version, including special features used (eg, explosion) | Google Chrome |
| Use of hand searching (eg, reference lists of obtained articles) | Data Sources and Searches |
| List of citations located and those excluded, including justification | Figure 1. Search plot diagram. |
| Method of addressing articles published in languages other than English | Data Sources and Searches |
| Method of handling abstracts and unpublished studies | Methods |
| Description of any contact with authors | Data Extraction and Quality Assessment |
| **Reporting of methods should include** | |
| Description of relevance or appropriateness of studies assembled for assessing the hypothesis to be tested | Methods |
| Rationale for the selection and coding of data (eg, sound clinical principles or convenience) | Study Selection |
| Documentation of how data were classified and coded (eg, multiple raters, blinding, and interrater reliability) | Methods |
| Assessment of confounding (eg, comparability of cases and controls in studies where appropriate) | Methods |
| Assessment of study quality, including blinding of quality assessors; stratification or regression on possible predictors of study results | Data Extraction and Quality Assessment |
| Assessment of heterogeneity | Data Synthesis and Analysis |
| Description of statistical methods (eg, complete description of fixed or random effects models, justification of whether the chosen models account for predictors of study results, dose-response models, or cumulative meta-analysis) in sufficient detail to be replicated | Data Synthesis and Analysis |
| Provision of appropriate tables and graphics | Methods and Results |
| **Reporting of results should include** | |
| Graphic summarizing individual study estimates and overall estimate | Figure 3 |
| Table giving descriptive information for each study included | Table 1 |
| Results of sensitivity testing (eg, subgroup analysis) | Results(Quantitative data synthesis, Heterogeneity analysis and sensitivity analysis) |
| Indication of statistical uncertainty of findings | Discussion |
| **Reporting of discussion should include** | |
| Quantitative assessment of bias (eg, publication bias) | Discussion |
| Justification for exclusion (eg, exclusion of non–English-language citations) | Discussion |
| Assessment of quality of included studies | Discussion |
| **Reporting of conclusions should include** | |
| Consideration of alternative explanations for observed results | Discussion |
| Generalisation of the conclusions (ie, appropriate for the data presented and within the domain of the literature review) | Discussion |
| Guidelines for future research | Discussion |
| Disclosure of funding source | Funding |
